# Clinical benefit of treatment after trastuzumab emtansine for HER2-positive metastatic breast cancer: a real-world multi-centre cohort study in Japan (WJOG12519B)

**DOI:** 10.1007/s12282-020-01192-y

**Published:** 2021-01-02

**Authors:** Takamichi Yokoe, Sasagu Kurozumi, Kazuki Nozawa, Yukinori Ozaki, Tetsuyo Maeda, Shu Yazaki, Mai Onishi, Akihiro Fujimoto, Sayuka Nakayama, Yuko Tsuboguchi, Tsutomu Iwasa, Hitomi Sakai, Misato Ogata, Mitsuo Terada, Meiko Nishimura, Takuma Onoe, Jun Masuda, Michiko Kurikawa, Hirotsugu Isaka, Kanako Hagio, Akihiko Shimomura, Yuta Okumura, Manabu Futamura, Mototsugu Shimokawa, Toshimi Takano

**Affiliations:** 1 Department of Breast Surgery, National Cancer Center Hospital East, Kashiwa, Japan; 2 Department of General Surgical Science, Gunma University Graduate School of Medicine, Maebashi, Japan; 3 Department of Clinical Oncology, Breast Oncology, Aichi Cancer Center Hospital, Nagoya, Japan; 4 Department of Medical Oncology, Toranomon Hospital, 2-2-2 Toranomon, Minato-ku, Tokyo, 105-8470 Japan; 5 Department of Surgical Oncology, Breast Oncology Center, Cancer Institute Hospital of the Japanese Foundation for Cancer Research, Tokyo, Japan; 6 Department of Breast and Medical Oncology, National Cancer Center Hospital, Tokyo, Japan; 7 Department of Breast Surgery, Tokyo Metropolitan Cancer and Infectious Diseases Center Komagome Hospital, Tokyo, Japan; 8 Breast Oncology Service, Saitama Medical University International Medical Center, Hidaka, Japan; 9 Advanced Cancer Translational Research Institute, Department of Surgery, Division of Breast Surgical Oncology, Showa University, Tokyo, Japan; 10 Department of Medical Oncology, Osaka City General Hospital, Osaka, Japan; 11 Department of Medical Oncology, Kindai University Faculty of Medicine, Osaka-sayama, Japan; 12 Department of Medical Oncology, Kobe City Hospital Organization Kobe City Medical Center General Hospital, Kobe, Japan; 13 Department of Breast Surgery, Nagoya City University Graduate School of Medical Sciences, Nagoya, Japan; 14 Department of Medical Oncology, Hyogo Cancer Center, Akashi, Japan; 15 Department of Breast and Endocrine Surgery, Toranomon Hospital, Tokyo, Japan; 16 Department of Breast Surgery, School of Medicine, Kyorin University, Tokyo, Japan; 17 Department of Breast Surgery, Hokkaido University Hospital, Sapporo, Japan; 18 Department of Breast and Medical Oncology, National Center for Global Health and Medicine, Tokyo, Japan; 19 Department of Internal Medicine, Kyushu University Beppu Hospital, Oita, Japan; 20 Department of Surgical Oncology, Graduate School of Medicine, Gifu University, Gifu, Japan; 21 Department of Biostatistics, Yamaguchi University Graduate School of Medicine, Yamaguchi, Japan

**Keywords:** Anti-HER2, Metastatic, Trastuzumab emtansine, Real world

## Abstract

**Background:**

Trastuzumab emtansine (T-DM1) treatment for human epidermal growth factor receptor-2 (HER2)-positive metastatic breast cancer after taxane with trastuzumab and pertuzumab is standard therapy. However, treatment strategies beyond T-DM1 are still in development with insufficient evidence of their effectiveness. Here, we aimed to evaluate real-world treatment choice and efficacy of treatments after T-DM1 for HER2-positive metastatic breast cancer.

**Methods:**

In this multi-centre retrospective cohort study involving 17 hospitals, 325 female HER2-positive metastatic breast cancer patients whose post-T-DM1 treatment began between April 15, 2014 and December 31, 2018 were enrolled. The primary end point was the objective response rate (ORR) of post-T-DM1 treatments. Secondary end points included disease control rate (DCR), progression-free survival (PFS), time to treatment failure (TTF), and overall survival (OS).

**Results:**

The median number of prior treatments of post-T-DM1 treatment was four. The types of post-T-DM1 treatments included (1) chemotherapy in combination with trastuzumab and pertuzumab (*n* = 102; 31.4%), (2) chemotherapy concomitant with trastuzumab (*n* = 78; 24.0%), (3), lapatinib with capecitabine (*n* = 63; 19.4%), and (4) others (*n* = 82; 25.2%). ORR was 22.8% [95% confidence interval (CI): 18.1–28.0], DCR = 66.6% (95% CI 60.8–72.0), median PFS = 6.1 months (95% CI 5.3–6.7), median TTF = 5.1 months (95% CI 4.4–5.6), and median OS = 23.7 months (95% CI 20.7–27.4).

**Conclusion:**

The benefits of treatments after T-DM1 are limited. Further investigation of new treatment strategies beyond T-DM1 is awaited for HER2-positive metastatic breast cancer patients.

**Supplementary Information:**

The online version contains supplementary material available at 10.1007/s12282-020-01192-y.

## Introduction

Recent developments in diagnostic accuracy and drug therapies have improved the outcomes of early-stage breast cancer patients [1, 2]. However, ~ 20% of breast cancer patients have a poor prognosis due to metastasis [3–5]. Metastatic spread has been estimated to be the primary cause of 90% of cancer-related deaths [6]. In routine clinical practice, the treatment regimens for metastatic breast cancer are determined based on a combination of immunohistochemical analyses of oestrogen receptor (ER), progesterone receptor (PgR), and human epidermal growth factor 2 (HER2) expression [7–9]. HER2-positive metastatic breast cancer was previously associated with a worse prognosis due to aggressive tumour progression and shorter patient survival [10]. However, the treatment strategy of HER2-overexpressing metastatic breast cancer changed drastically after trastuzumab was approved by the Food and Drug Administration (FDA) in 1998 [11–13]. Following this, additional HER2-targeted agents, such as lapatinib, pertuzumab, and trastuzumab emtansine (T-DM1), were approved for the treatment of HER2-positive metastatic breast cancers [14]. In 2019–2020, FDA also approved trastuzumab deruxtecan (T-Dxd), tucatinib, and neratinib for HER2-positive metastatic breast cancer.

T-DM1 is an antibody–drug conjugate that incorporates the effectiveness of trastuzumab with the cytotoxic activity of the microtubule-inhibitory agent emtansine (DM1). T-DM1 inhibits the HER2 signalling pathway, resulting in cessation of cancer cell proliferation. Moreover, the use of T-DM1 avoids the exposure of chemotherapy to normal tissues by targeting chemotherapy delivery, specifically to HER2-overexpressing cancer cells. Recent clinical trials indicate that T-DM1 is useful for the treatment of HER2-positive metastatic breast cancer patients. In the EMILIA trial that assessed the efficacy of T-DM1 in HER2-positive metastatic breast cancer, T-DM1 significantly prolonged progression-free and overall survival with less toxicity compared with lapatinib plus capecitabine in patients previously treated with trastuzumab and a taxane [15]. Results from the MARIANNE study involving patients with HER2-positive metastatic breast cancer showed that the median response duration was 12.5 months in patients who were treated with trastuzumab plus taxane, 20.7 months in those treated with T-DM1, and 21.2 months in patients treated with T-DM1 plus pertuzumab [16].

Since T-DM1 was approved as a new agent based on these clinical studies in 2014, the use of T-DM1 for HER2-positive metastatic breast cancer after taxane with trastuzumab and pertuzumab has been established as standard therapy. However, treatment strategies beyond T-DM1 are still in development as there is insufficient evidence regarding their current status in this setting. In this multi-centre cohort study, we aimed to clarify the real-world treatment choice and efficacy of treatments after T-DM1 for HER2-positive metastatic breast cancer.

## Patients and methods

### Study design

This study was an observational retrospective multi-centre cohort study involving 17 hospitals which participated in the West Japan Oncology Group (WJOG). The study was registered in the UMIN Clinical Trial Registry (UMIN000037747) and approved by the ethics committee in all 17 sites. Data from each facility were collected by the data centre at WJOG using an electronic data capture system.

HER2-positive metastatic breast cancer patients who had received T-DM1 were included in this trial. We defined the treatments after treatment with T-DM1 as “post-T-DM1 treatments.” The main eligibility criteria were as follows: (1) metastatic breast cancer, (2) HER2-positive breast cancer, and (3) patients whose post-T-DM1 treatments initiated between January 1, 2014 and December 31, 2018. Patients who received investigational drugs as the post-T-DM1 treatment were excluded from the current study. ER and PgR are considered positive if 1% or more of the tumour cells demonstrate positive nuclear staining by immunohistochemistry. The definition of HER2 positive is “HER2 3 + ” or “HER2 ISH positive”.

### Outcomes of interest

The analysis was designed to evaluate clinical backgrounds and the effectiveness of post-T-DM1 treatments in HER2-positive metastatic breast cancer patients in practice in Japan. The primary end point was objective response rate (ORR), and secondary end points included disease control rate (DCR), progression-free survival (PFS), time to treatment failure (TTF), and overall survival (OS).

The ORR is the percentage of patients with a complete response (CR) or partial response (PR) in the best overall response. The DCR is the proportion of patients with CR, PR, or stable disease (SD) in the best overall response. PFS was calculated starting from the start date of the treatment and ending with the earliest event of disease progression or death. Deterioration of the disease was defined as the day clinically judged as progressive disease (PD), regardless of imaging. The therapeutic effect was mainly calculated following the response evaluation criteria in solid tumours (RECIST), with an additional aid by the attending physician. TTF was calculated starting from the start date of the target treatment and ending with the earliest event of disease progression or treatment discontinuation. OS was defined as the time from the date of diagnosis until the last date for which survival status data were available (last follow-up date: June 31st, 2019). All deaths were considered events regardless of the reason.

The number of prior treatments was determined as follows. Single use of anti-HER2 drugs was not counted as a treatment line, whereas single use of endocrine therapy or chemotherapy was counted as one treatment line. T-DM1 was counted as one line of chemotherapy. Pre- and post-operative chemotherapies and endocrine therapies were not included.

### Statistical analysis

For patient backgrounds, frequencies and percentages were calculated for categorical data, and descriptive statistics values were calculated for quantitative data.

ORR and DCR were calculated for populations with target lesions, and 95% confidential intervals (CIs) were calculated based on the Clopper–Pearson method. PFS, TTF, and OS were calculated for the overall population, and the survival curves were estimated using the Kaplan–Meier method. The CIs for median survival and survival rates were calculated according to the Brookmeyer and Crowley method and Greenwood’s formula, respectively. The date of disease progression was defined as the date clinically determined as PD.

The subgroup analyses were pre-planned with the following parameters: T-DM1 resistant or refractory, Eastern Cooperative Oncology Group Performance Status (ECOG PS) of 0 or 1, the presence or absence of brain metastases, age under 65 or 65 and up, best overall response for T-DM1 CR/PR/SD or PD, prior therapy with or without pertuzumab, positive or negative ER and PgR status, the presence or absence of visceral metastases at the start of post-T-DM1 treatment, and the number of treatment lines before T-DM1 of zero and one or two or more. “Resistant” was defined as cases that once showed SD, PR, or CR but eventually resulted in PD. The category “refractory” was defined as cases in which the response of T-DM1 treatment was PD from the beginning. Visceral metastases refer to liver or lung/pleural metastases.

Statistical significance was defined as a p value less than 0.05. Statistical analyses were performed using SAS version 9.4 (SAS Institute, North Carolina, USA).

## Results

### Patient characteristics

**Fig. 1:**
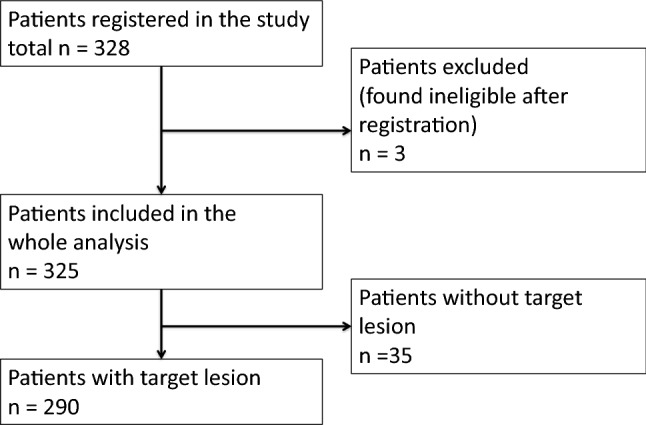
Flow diagram of the patient population

A flow diagram of the included patients is shown in Fig. 1. Of the 328 enrolled patients, 325 patients were included in the final analysis. Of all cases analysed, 290 patients had a target lesion (89.2%).

A summary of patient characteristics is shown in Table 1 and Supplemental Table 1. All patients were female, and the median age was 59 years (range 25–96). Of the 325 patients, there were 182 (56%) ER-positive cases. The number of patients with de novo stage IV was 124 (38.2%), and 201 (61.8%) experienced recurrence. There were 273 (84%) patients with an ECOG PS of 0 or 1 at the start of post-T-DM1 therapy. Brain metastases occurred in 61 cases (18.8%). The number of patients previously treated with pertuzumab, taxane, or anthracycline was 217 (66.8%), 279 (85.8%), and 102 (31.4%), respectively. The median line of post-T-DM1 treatment was 4 (range 2 to more than 10).

**Table 1:** Patient characteristics of eligible cohort (*n* = 325)

Characteristics	Number of cases (*n* = 325)	%
Sex		
Male	0	0.0%
Female	325	100.0%
Age	Median (min—max)	59 (25–96)
< 40	19	5.8%
40–64	200	61.5%
65–74	74	22.8%
75 or more	32	9.8%
Menopause		
Pre	39	12.0%
Post	230	70.8%
Unknown	56	17.2%
Stage		
De novo Stage IV	124	38.2%
Recurrence	201	61.0%
Operation		
Yes	230	70.8%
No	95	29.2%
ER		
Positive	182	56.0%
Negative	143	44.0%
Unknown	0	0.0%
PgR		
Positive	114	35.1%
Negative	209	64.3%
Unknown	2	0.6%
HER2 score		
0	0	0.0%
1 +	1	0.3%
2 +	77	23.7%
3 +	246	75.7%
Missing value	1	0.3%
HER2 ISH		
−	5	1.5%
+	87	26.8%
Unknown	233	71.7%
Pertuzumab use in treatment before T-DM1		
Yes	217	66.8%
No	108	33.2%
Taxane use in treatment before T-DM1		
Yes	279	85.8%
No	46	14.2%
Anthracycline use in treatment before T-DM1		
Yes	102	31.4%
No	223	68.6%
Number of treatment before T-DM1	median (min–max)	2 (0–≥ 10)
0 or 1	120	36.9%
2 or more	205	63.1%
PS at starting post-T-DM1 treatment		
0	102	31.4%
1	171	52.6%
2 or more	18	5.5%
Unknown	34	10.5%
Presence of metastasis		
Yes	314	96.6%
No	11	3.4%
Metastatic cites (multiple selection allowed)		
Liver	110	33.8%
Lung or pleural	162	49.8%
Brain	61	18.8%
Others	224	68.9%
Presence of target lesion		
Yes	290	89.2%
No	35	10.8%

### Clinical benefit of post-T-DM1 treatment

We analysed ORR and DCR in 290 cases with a target lesion (Table 2). The ORR was 22.8% (95% CI 18.1–28.0%), and DCR was 66.6% (60.8–72.0%). CR occurred in 0.7% of patients.

**Table 2:** ORR and DCR of post T-DM1 (*n* = 290)

	Number of cases (*n* = 290)	%	95% CI
CR	2	0.7	
PR	64	22.1	
SD	127	43.8	
PD	81	27.9	
NE	16	5.5	
ORR	66	22.8	(18.1–28.0)
DCR	193	66.6	(60.8–72.0)

Treatment line: median 4 (2–≥ 10)

*CR* complete response, *PR* partial response, *SD* stable disease, *PD* progressive disease, *NE* not evaluable, *ORR* overall response rate, *DCR* disease control rate, *95% CI* 95% confidence interval

**Fig. 2:**
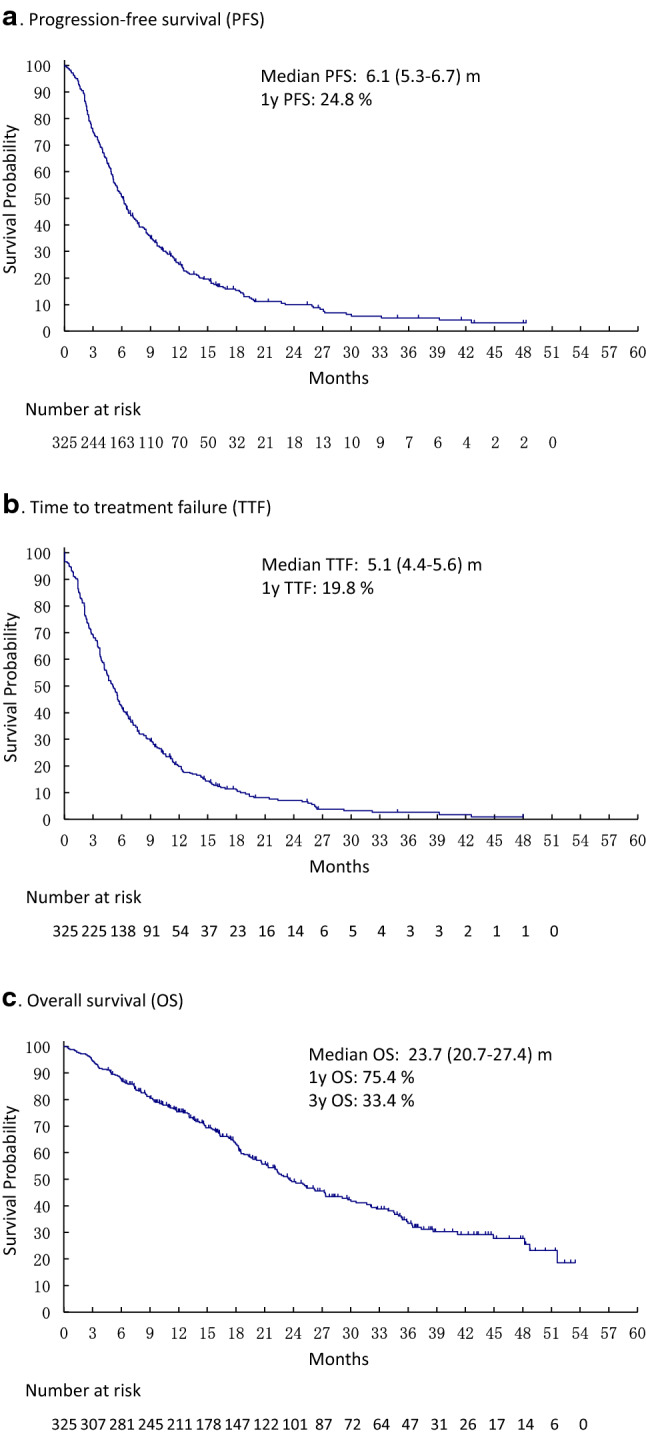
Survival curves of post-T-DM1 treatments (*n* = 325). **a** Progression-free survival (PFS) **b** Time to treatment failure (TTF). **c** Overall survival (OS)

Survival was analysed in the entire population (*n* = 325) with a median follow-up time of 16.3 months (95% CI 14.3–18.2). Median PFS was 6.1 months (95% CI 5.3–6.7) with a 1-year PFS rate of 24.8% (Fig. 2a). The median TTF was 5.1 months (95% CI 4.4–5.6) with a 1-year TTF rate of 19.8% (Fig. 2b). Median OS was 23.7 months (95% CI 20.7–27.4) with a 1-year OS rate of 75.4% and 3-year OS rate of 33.4% (Fig. 2c).

The selected post-T-DM1 treatment was as follows (Table 3 and Supplemental Table 2): (1) chemotherapy in combination with trastuzumab and pertuzumab (*n* = 102; 31.4%), (2) chemotherapy concomitant with trastuzumab only (*n* = 78; 24.0%), (3) lapatinib with capecitabine (*n* = 63; 19.4%), and (4) others (*n* = 82; 25.2%). Chemotherapy used with trastuzumab and pertuzumab in descending order were taxane, eribulin, and vinorelbine, while chemotherapies used with trastuzumab only included gemcitabine and capecitabine. Anthracycline was selected in 17 cases (5.2%). Of 217 cases with previous pertuzumab use, pertuzumab was administered again in 67 cases as post-T-DM1 treatment.

**Table 3:** Types of post T-DM1 treatment (*n* = 325)

	Number of cases (*n* = 325)	%
Lapatinib + capecitabine	63	19.4%
Trastuzumab + pertuzumab + CTx	102	31.4%
Trastuzumab + pertuzumab + HTx	3	0.9%
Trastuzumab + pertuzumab	2	0.6%
Trastuzumab + CTx	78	24.0%
Trastuzumab + HTx	10	3.1%
Trastuzumab	11	3.4%
Anthracycline	17	5.2%
other CTx	24	7.4%
HTx	6	1.8%
Others	9	2.8%

*CTx* chemotherapy, *HTx* hormonal therapy

To determine the effectiveness of each treatment, we evaluated the following three groups individually: chemotherapy concomitant with trastuzumab and pertuzumab, chemotherapy concomitant with trastuzumab only, and lapatinib with capecitabine. Patients treated with chemotherapy in combination with trastuzumab and pertuzumab showed the highest ORR of 27.1% (95% CI 18.5–37.1), while ORR of chemotherapy in combination with trastuzumab, and lapatinib with capecitabine were 17.9% (95% CI 9.6–29.2) and 20.0% (95% CI 10.4–33.0), respectively. Those treated with lapatinib with capecitabine showed the highest DCR of 74.5% (95% CI 60.1–85.3) due to the percentage of the SD population accounting for more than 50%, whereas DCR of chemotherapy in combination with trastuzumab and pertuzumab, and chemotherapy in combination with trastuzumab were 67.7% (95% CI 57.4.6–76.9) and 58.2% (95% CI 45.5–70.2), respectively. The survival curves of each treatment were almost identical (Supplemental Fig.1). Furthermore, the OS survival curves were slightly worse in the lapatinib with capecitabine group. Detailed patient backgrounds showed that patients with T-DM1 best response PD were 39.7% in lapatinib with capecitabine, 31.4% in chemotherapy in combination with trastuzumab and pertuzumab, and 30.8% in chemotherapy in combination with trastuzumab. As for patients with brain metastasis, these were 31.7%, 14.7%, and 15.4%, respectively (Data not shown).

### Use of post-T-DM1 treatment in subgroup analysis

**Fig. 3:**
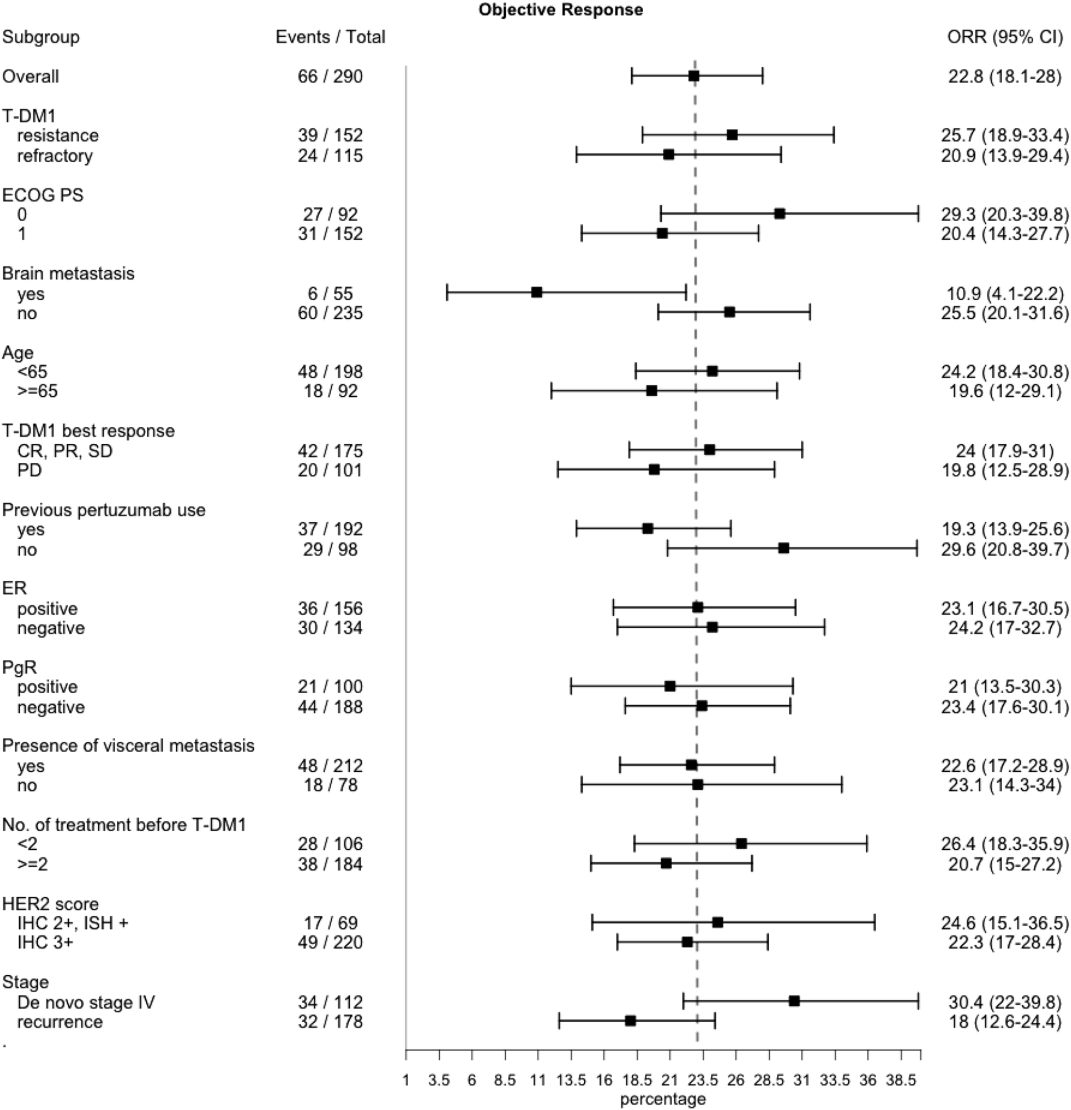
Subgroup analysis of ORR (*n* = 290)

Prespecified subgroup analyses of ORR showed consistent results across subgroups as follows (Fig. 3): age (younger than 65 = 24.2%, 65 or older = 19.6%), response to T-DM1 (CR/PR/SD = 24.0%, PD = 19.8%), T-DM1 best response (resistance = 25.7%, refractory = 20.9%), ER status (positive = 23.1%, negative = 24.2%), PgR status (positive = 21%, negative = 23.4%), the presence of visceral metastasis (with metastasis = 22.6%, without metastasis = 23.1%), and treatment line before T-DM1 (fewer than two = 26.4%, two or more = 20.7%). Subgroup analyses of TTF and PFS also showed consistent results across all subgroups (Supplemental Fig. 3 and 4). Patients with previous pertuzumab use showed similar results (ORR = 19.3%, DCR = 64.1%, PFS = 5.9 months, TTF = 4.6 months, and OS = 23 months) with those without previous pertuzumab use (ORR = 29.6%, DCR = 71.4%, PFS = 6.5 months, TTF = 5.6 months, and OS = 24.2 months). Subgroup analyses of pertuzumab rechallenge consisting of 217 cases with previous pertuzumab use showed the following results: the pertuzumab rechallenge group (ORR = 19%, DCR = 63.5%, PFS = 5.5 months, TTF = 4.6 months, and OS = 29.7 months) and the pertuzumab non-rechallenge group (ORR = 19.4%, DCR = 64.3%, PFS = 4.5 months, TTF = 6.0 months, and OS = 22.1 months) (Table 4).

**Table 4:** Effects of pertuzumab rechallenge in post T-DM1 treatment (ORR and DCR, *n* = 192, TTF, PFS, and OS, *n* = 217)

Response	Pertuzumab rechallenge (*n* = 63)			Pertuzumab non-rechallenge (*n* = 129)		
	Events/total	%	95% CI	Events/total	%	95% CI
ORR	12/63	19	(10.2–30.9)	25/129	19.4	(13.0–27.3)
DCR	40/63	63.5	(50.4–75.3)	83/129	64.3	(55.4–72.6)

Survival	Pertuzumab rechallenge (*n* = 67)			Pertuzumab non-rechallenge (*n* = 150)		
	Events/total	Months	95% CI	Events/total	Months	95% CI
TTF	56/67	4.6	(3.0–7.8)	141/150	4.5	(3.7–5.8)
PFS	54/67	5.5	(4.4–10.2)	133/150	6	(4.9–6.7)
OS	27/67	29.7	(18.8–51.6)	82/150	22.1	(18.3–26.3)

*ORR* overall response rate, *DCR* disease control rate, *TTF* time to treatment failure, *PFS* progressive-free survival, *OS* overall survival, *95% CI* 95% confidence interval. ORR and DCR were calculated for populations with target lesions, and 95% confidential intervals (CIs) were calculated based on the Clopper–Pearson method. PFS, TTF, and OS were calculated for the overall population, and the survival curves were estimated using the Kaplan–Meier method. The CIs for median survival and survival rates were calculated according to the Brookmeyer and Crowley method and Greenwood’s formula, respectively

Some trends were observed in subgroup analyses. Regarding ORR, patients without brain metastasis showed ORR of 25.5% (95% CI 20.1–31.6), as compared with 10.9% (95% CI 4.1–22.2) in cases with brain metastasis. Patients with de novo stage IV breast cancer showed ORR of 30.4% (95%CI: 22–39.8), while those with recurrence showed 18.0% (95% CI 12.6–24.4) (Fig. 3). Subgroup analyses of DCR, TTF, PFS, and OS are shown in Supplemental Fig. 2–5.

## Discussion

In this study, we evaluated the treatment choice and efficacy of post-T-DM1 treatments in real-world practice. We found that chemotherapy in combination with trastuzumab and pertuzumab was the most commonly selected post-T-DM1 treatment, and the efficacy of post-T-DM1 treatment was relatively limited (ORR = 22.8%, PFS = 6.1 months, and OS = 23.7 months). This study results provided insights into understanding the current practice patterns for patients who had prior treatment with T-DM1.

The efficacy of post-T-DM1 treatment in this study is lower than that of T-DM1 as the second-line therapy for HER2-positive metastatic breast cancer in the EMILIA study (ORR = 43.6%, PFS = 9.6 months, OS = 30.9 month). With a very limited effect of PFS at 6 months, third-line treatment for HER2-positive breast cancer requires further improvement. As will be described later, the development of new drugs is being carried out for this purpose, and promising drugs are appearing. Second, regarding the subgroup analysis, some trends seen in the results of this study are consistent with the results of other HER2-positive metastatic breast cancer trials. Greater ORR can be expected with patients without brain metastasis, and patients with de novo stage IV. Greater DCR can be expected when the prior T-DM1 resulted in a good response. With better PS and response to prior T-DM1 therapy, patients may likely achieve longer overall survival. Although the effect of third-line treatment is limited, it is expected that the current treatment is to some extent efficient on patients in these subgroups. Third, the patients in the group of laptainib with capecitabine as post-T-DM1 treatment tended to have inferior OS. Based on the patient background, it is surmised that this is due to two factors: that there were many cases with (1) low response to prior T-DM1, and (2) brain metastasis.

At least two implications from the subgroup analyses can be pointed out. First, previous pertuzumab use may not affect the effect of post-T-DM1. In our study, previous pertuzumab use did not affect ORR, DCR, PFS, TTF and OS. In post hoc analysis of T-DXd, previous pertuzumab use did not affect the results either [17]. But the possibility that treatment after pertuzumab can be less effective has been suggested [18–20], though it was based on small and observational studies examining the efficacy of T-DM1 in patients with previous pertuzumab. Thus, further investigation is warranted to evaluate the influence of previous pertuzumab use over post-T-DM1 treatment.

Second, we examined the effect of pertuzumab rechallenge. At this time, there is no evidence of the rechallenge effect of pertuzumab, and the guidelines do not recommend rechallenge. All results of the subgroup analyses were consistent between the pertuzumab rechallenge group and non-rechallenge group. OS tends to be better in pertuzumab rechallenge group, though evaluation in a prospective study is necessary. The results of an ongoing multicentre randomized phase-three study examining the effect of pertuzumab rechallenge are awaited (PRECIOUS study: UMIN000018202) [21]. For the above two points, we are planning additional post hoc analysis using the propensity score matching method.

There has recently been remarkable progress in the development of new drugs for HER2-positive metastatic breast cancer [22]. These include tyrosine kinase inhibitors (neratinib and tucatinib), novel antibody–drug conjugates (trastuzumab-deruxtecan; T-DXd), novel anti-HER2 antibodies (margetuximab), and combination therapies of immune checkpoint inhibitors and anti-HER2 drugs. Some of these agents are primarily being developed for the treatment after T-DM1. DESTINY-Breast01 [23], HER2CLIMB [24], NALA [25], and SOPHIA [26] clinical trials of T-DXd, tucatinib, neratinib, and margetuximab, respectively, have recently reported promising results. By offering real-world evidence on the current use of post-T-DM1 therapies, our results provide an opportunity for the reinterpretation of studies and implications for the understanding of these four studies.

The results of the current study complement the DESTINY-Breast01 trial, which lacked a control arm. The patients enrolled in the DESTINY trial had prior T-DM1 and had median of six lines of previous treatments. Regardless of the heavily treated population, the study showed markedly positive results, including an ORR of 60.9%, DCR of 97.3%, and PFS of 16.4 months. Although a direct comparison is not readily applicable, higher response rate and longer PFS have been shown despite the heavily treated and less treatable population background compared with our study; thus the advantage of T-DXd has been more evident. T-DXd is a highly promising treatment, even though careful monitoring and management are still required for adverse events, such as interstitial pneumonia.

The patients in the intervention group in HER2CLIMB received tucatinib with trastuzumab and capecitabine. Adding tucatinib to trastuzumab and capecitabine resulted in better PFS and OS outcomes than adding a placebo. Specifically, the ORR was 41% vs. 23%, PFS was 7.8 vs. 5.6 months, and OS was 21.9 vs. 17.4 months at a median of three previous treatments. PFS of the control arm in HER2CLIMB was shorter than that of our study, which may be attributed to the large number of cases of brain metastasis included in this trial (18.8% in our study and ~ 46% in HER2CLIMB). Taking this into account, the ORR and PFS of HER2CLIMB are considered to be very high.

The NALA trial compared neratinib (an irreversible pan-HER tyrosine kinase inhibitor) plus capecitabine with lapatinib plus capecitabine in patients with stage IV HER2-positive metastatic breast cancer who had received two or more previous HER2-directed therapies. The ORR was 32.8% vs. 26.7%, and the median PFS and OS were 5.6 months vs. 5.5 and 21 months vs. 18.7, respectively. In the NALA trial, the number of patients who received T-DM1 and pertuzumab as prior anti-HER2 therapy was relatively small at 54% and 42%, respectively, which may account for the shorter prognosis in this trial.

The SOPHIA trial compared margetuximab plus chemotherapy with trastuzumab plus chemotherapy in HER2-positive metastatic breast cancer patients after one to three prior anti-HER2 therapies. Margetuximab is an Fc-modified anti-HER2 monoclonal antibody that showed enhanced antibody-dependent cell-mediated cytotoxicity compared with trastuzumab in in vitro studies. Margetuximab prolonged PFS (6.9 vs. 5.1 months) and showed a higher ORR (22% vs. 16%) compared with trastuzumab. Similar to trastuzumab, its safety was acceptable. Infusion-related reactions were more common with margetuximab, but these were manageable with premedication. Our studies differ in that all patients received pertuzumab as a pre-treatment in SOPHIA. The publication of this paper is highly anticipated, preferably with the details of other patient backgrounds.

Our results are consistent with the results of these trials in that all conventional treatments have low efficacy in terms of ORR, PFS. The current study adds to a positive perspective that new drugs are expected to prolong overall survival. Furthermore, our real-world evidence on the current use of post-T-DM1 treatments contributes to an in-depth understanding and renewed evaluation of preceding trials on promising drugs under development.

We recognized several limitations of this study. First, due to the retrospective nature of this study, heterogeneity in the patient population and differences in treatment choice are inevitable. Because of the absence of a comparative treatment group, only limited comparisons with prior clinical trial results are possible. Second, because this study collected clinical data from daily practice, there may have been differences in timing and tumour evaluation methods, which may have affected our results. Third, as for selection bias, countermeasures were taken to ensure that the patients were registered not only from cancer specialized institutions, but also from other non-specialized institutions, thereby reducing referral filter bias. Additionally, selection bias within institutions was reduced because all eligible cases were registered impartially. Fourth, no information on adverse events was collected.

In conclusion, we found that post-T-DM1 treatments showed modest anti-tumour activity. Further investigation of new treatment strategy beyond T-DM1 is awaited for HER2-positive metastatic breast cancer patients.

## Supplementary Information

Below is the link to the electronic supplementary material.

Supplementary file1 (PPTX 871 KB)

Supplementary file2 (DOCX 19 KB)
