# Supplementary material for: Clinical benefit of treatment after trastuzumab emtansine for HER2-positive metastatic breast cancer: a real-world multi-centre cohort study in Japan (WJOG12519B)
Source: Breast Cancer. 2021 Jan 2;28(3):581–91. doi: 10.1007/s12282-020-01192-y (PMC8064974; doi:10.1007/s12282-020-01192-y)
Supplement: Supplementary file 1 — Supplementary file1 (PPTX 871 KB) [file 12282_2020_1192_MOESM1_ESM.pptx]

## Slide 1
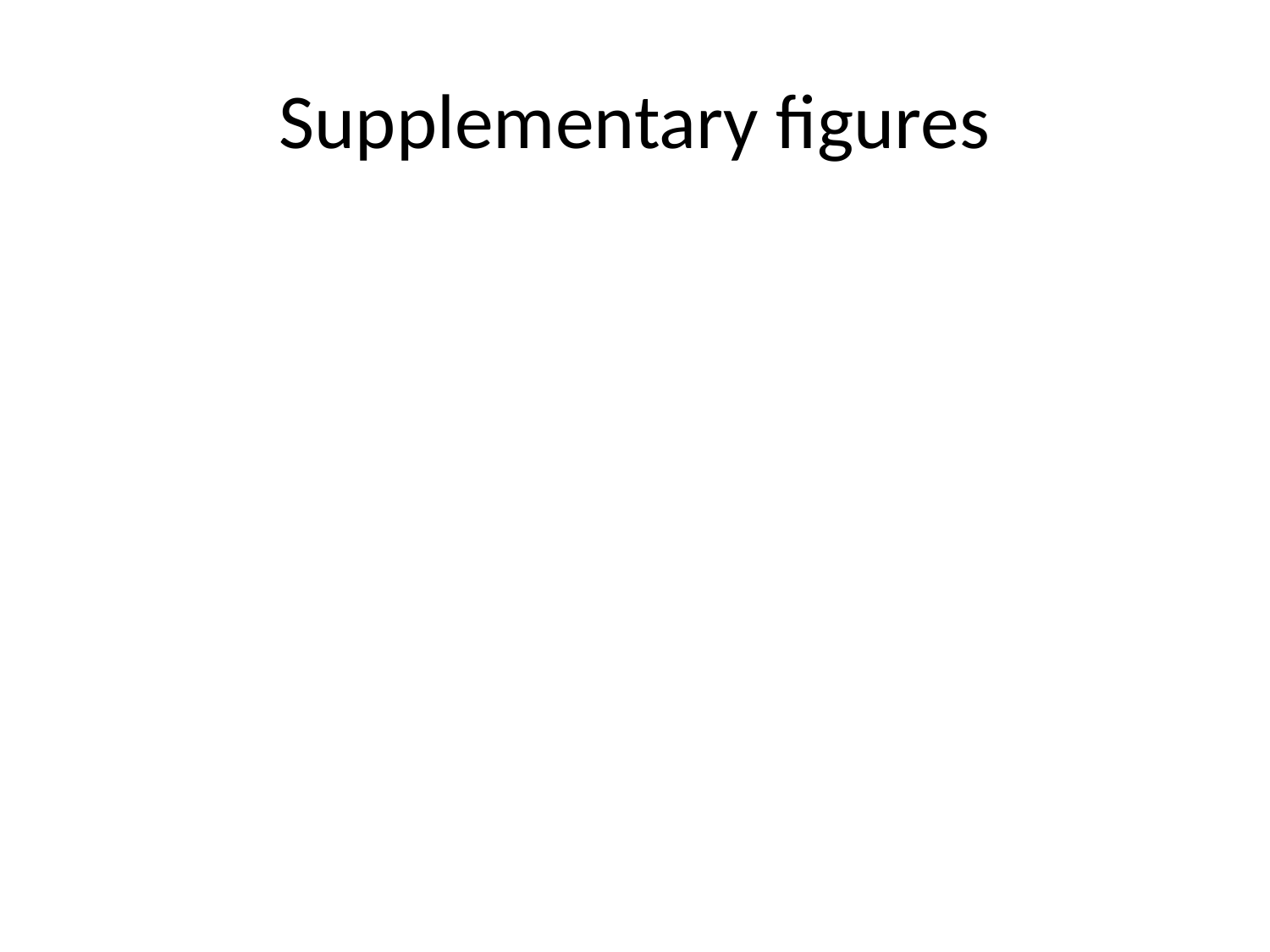

# Supplementary figures

## Slide 2
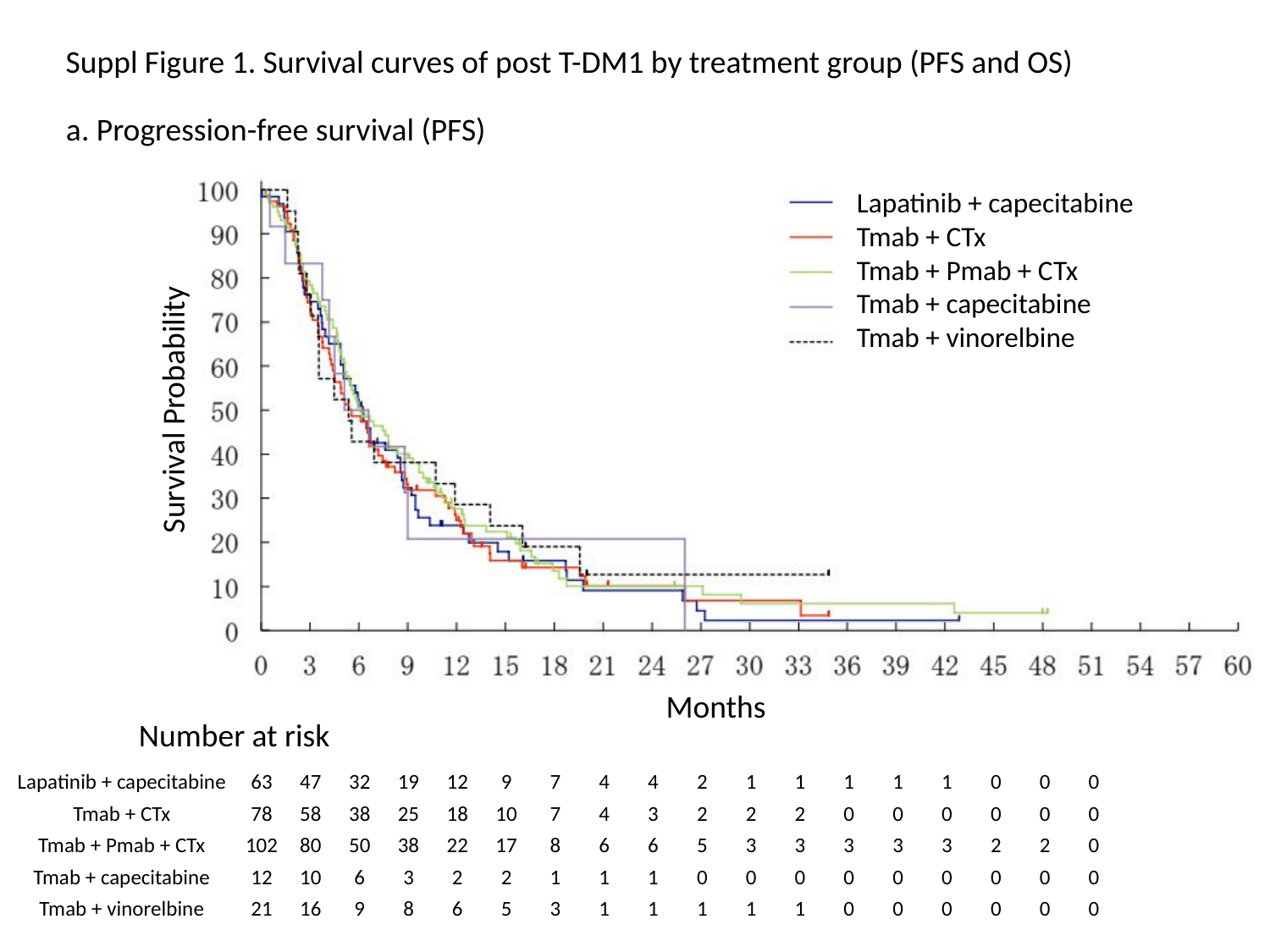

Suppl Figure 1. Survival curves of post T-DM1 by treatment group (PFS and OS)
a. Progression-free survival (PFS)
Lapatinib + capecitabine
Tmab + CTx
Tmab + Pmab + CTx
Tmab + capecitabine
Tmab + vinorelbine
Survival Probability
Months
Number at risk
| Lapatinib + capecitabine | 63 | 47 | 32 | 19 | 12 | 9 | 7 | 4 | 4 | 2 | 1 | 1 | 1 | 1 | 1 | 0 | 0 | 0 |
| --- | --- | --- | --- | --- | --- | --- | --- | --- | --- | --- | --- | --- | --- | --- | --- | --- | --- | --- |
| Tmab + CTx | 78 | 58 | 38 | 25 | 18 | 10 | 7 | 4 | 3 | 2 | 2 | 2 | 0 | 0 | 0 | 0 | 0 | 0 |
| Tmab + Pmab + CTx | 102 | 80 | 50 | 38 | 22 | 17 | 8 | 6 | 6 | 5 | 3 | 3 | 3 | 3 | 3 | 2 | 2 | 0 |
| Tmab + capecitabine | 12 | 10 | 6 | 3 | 2 | 2 | 1 | 1 | 1 | 0 | 0 | 0 | 0 | 0 | 0 | 0 | 0 | 0 |
| Tmab + vinorelbine | 21 | 16 | 9 | 8 | 6 | 5 | 3 | 1 | 1 | 1 | 1 | 1 | 0 | 0 | 0 | 0 | 0 | 0 |

## Slide 3
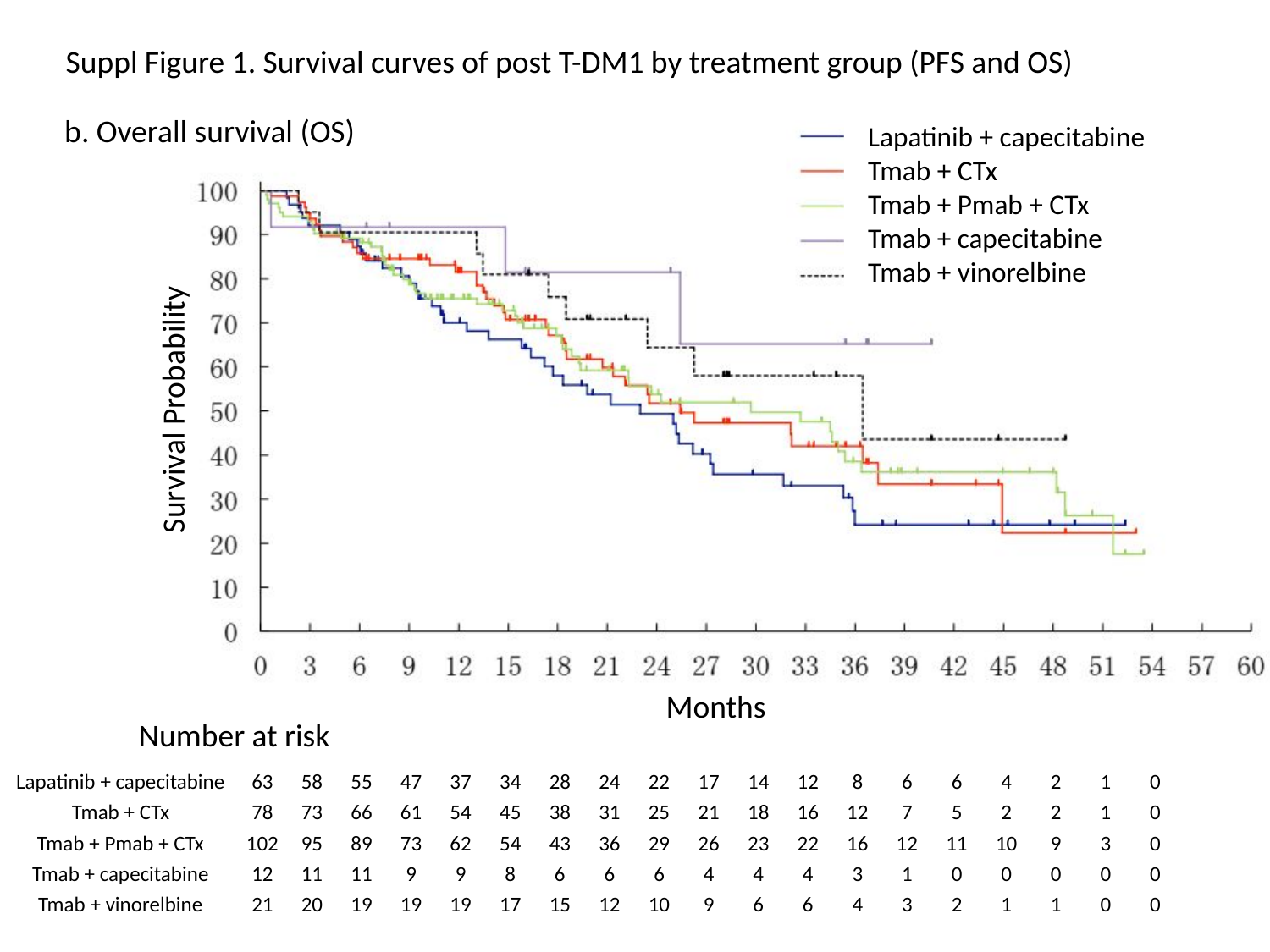

Suppl Figure 1. Survival curves of post T-DM1 by treatment group (PFS and OS)
b. Overall survival (OS)
Lapatinib + capecitabine
Tmab + CTx
Tmab + Pmab + CTx
Tmab + capecitabine
Tmab + vinorelbine
Survival Probability
Months
Number at risk
| Lapatinib + capecitabine | 63 | 58 | 55 | 47 | 37 | 34 | 28 | 24 | 22 | 17 | 14 | 12 | 8 | 6 | 6 | 4 | 2 | 1 | 0 |
| --- | --- | --- | --- | --- | --- | --- | --- | --- | --- | --- | --- | --- | --- | --- | --- | --- | --- | --- | --- |
| Tmab + CTx | 78 | 73 | 66 | 61 | 54 | 45 | 38 | 31 | 25 | 21 | 18 | 16 | 12 | 7 | 5 | 2 | 2 | 1 | 0 |
| Tmab + Pmab + CTx | 102 | 95 | 89 | 73 | 62 | 54 | 43 | 36 | 29 | 26 | 23 | 22 | 16 | 12 | 11 | 10 | 9 | 3 | 0 |
| Tmab + capecitabine | 12 | 11 | 11 | 9 | 9 | 8 | 6 | 6 | 6 | 4 | 4 | 4 | 3 | 1 | 0 | 0 | 0 | 0 | 0 |
| Tmab + vinorelbine | 21 | 20 | 19 | 19 | 19 | 17 | 15 | 12 | 10 | 9 | 6 | 6 | 4 | 3 | 2 | 1 | 1 | 0 | 0 |

## Slide 4
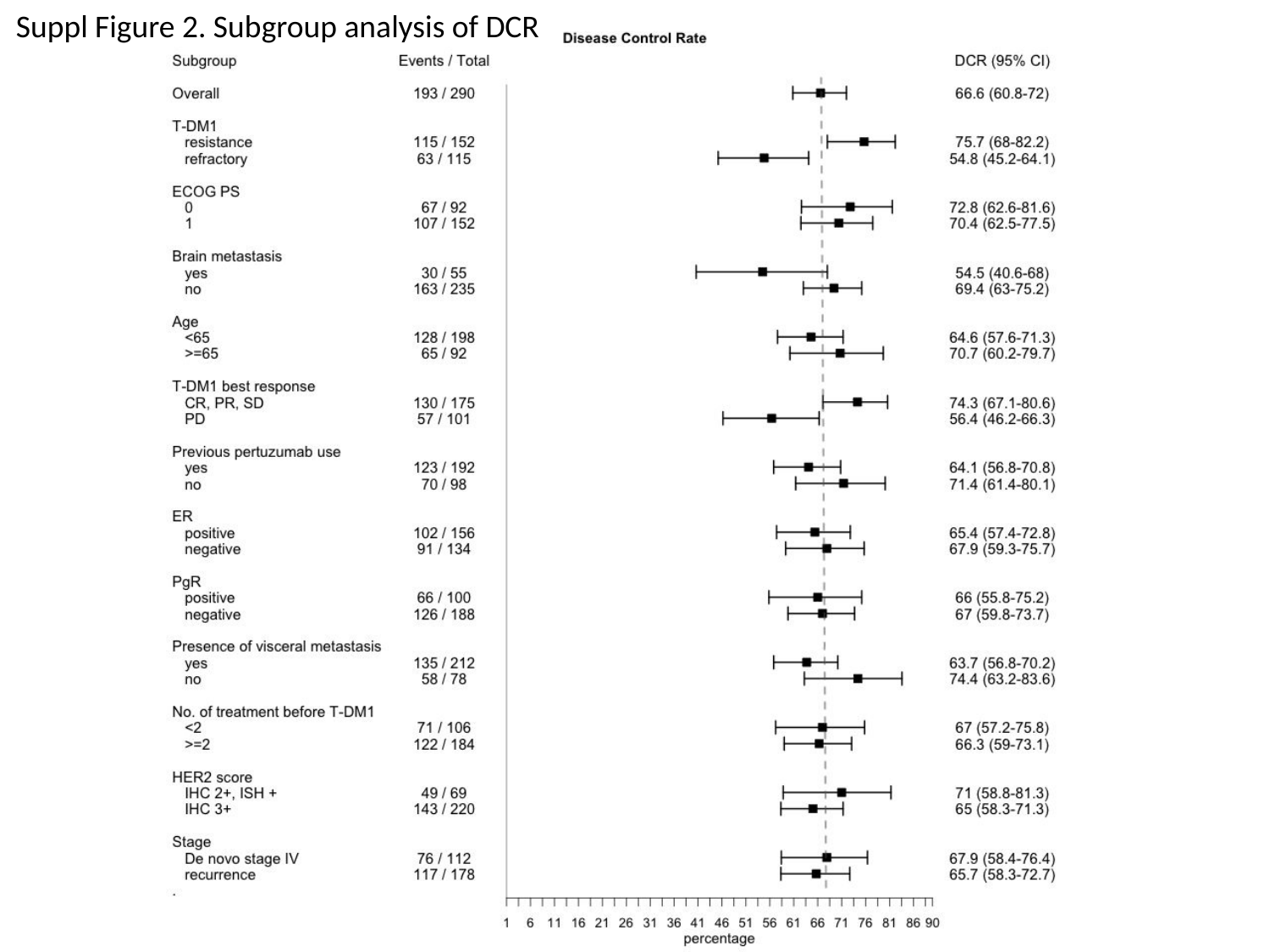

Suppl Figure 2. Subgroup analysis of DCR

## Slide 5
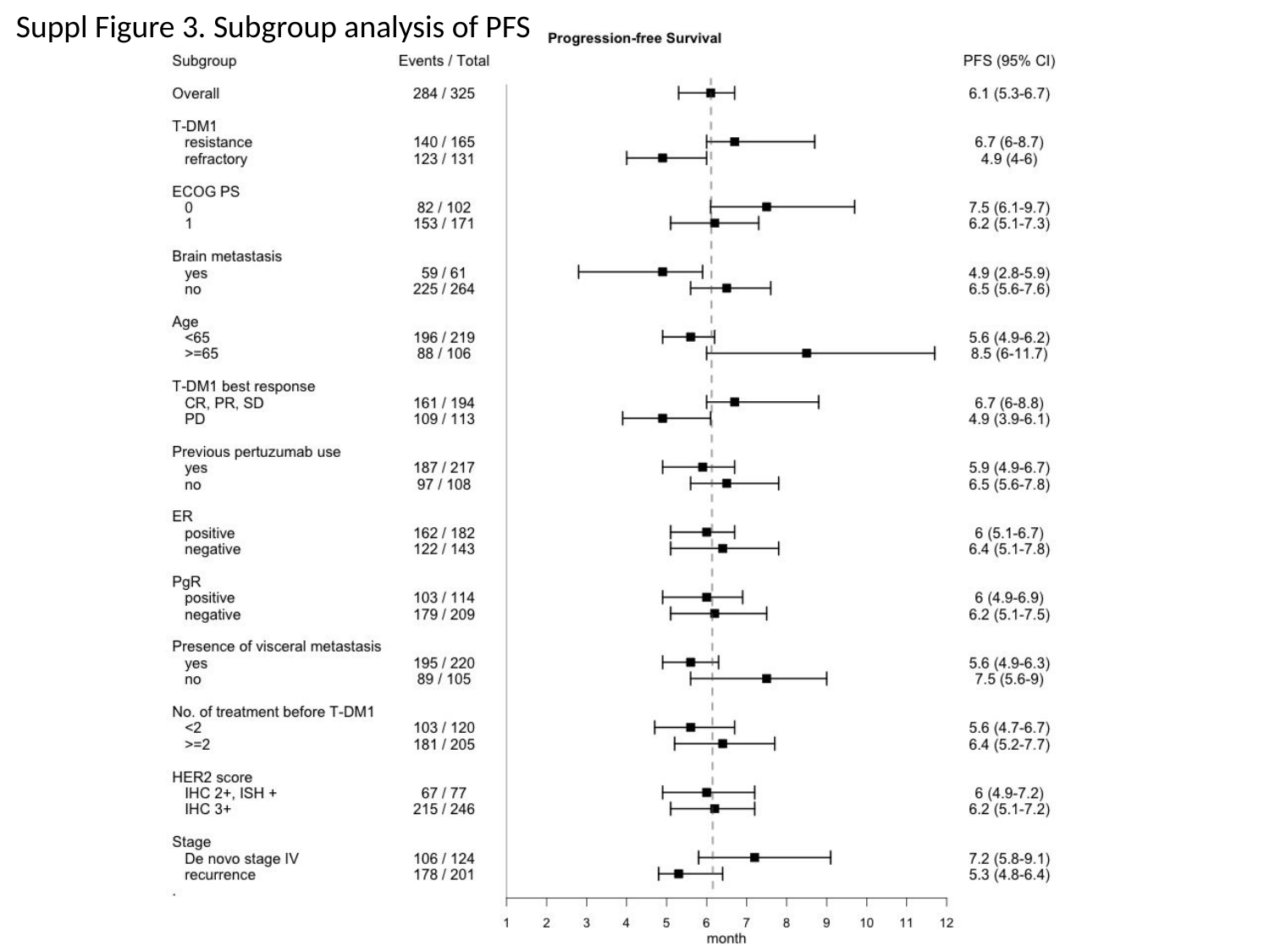

Suppl Figure 3. Subgroup analysis of PFS

## Slide 6
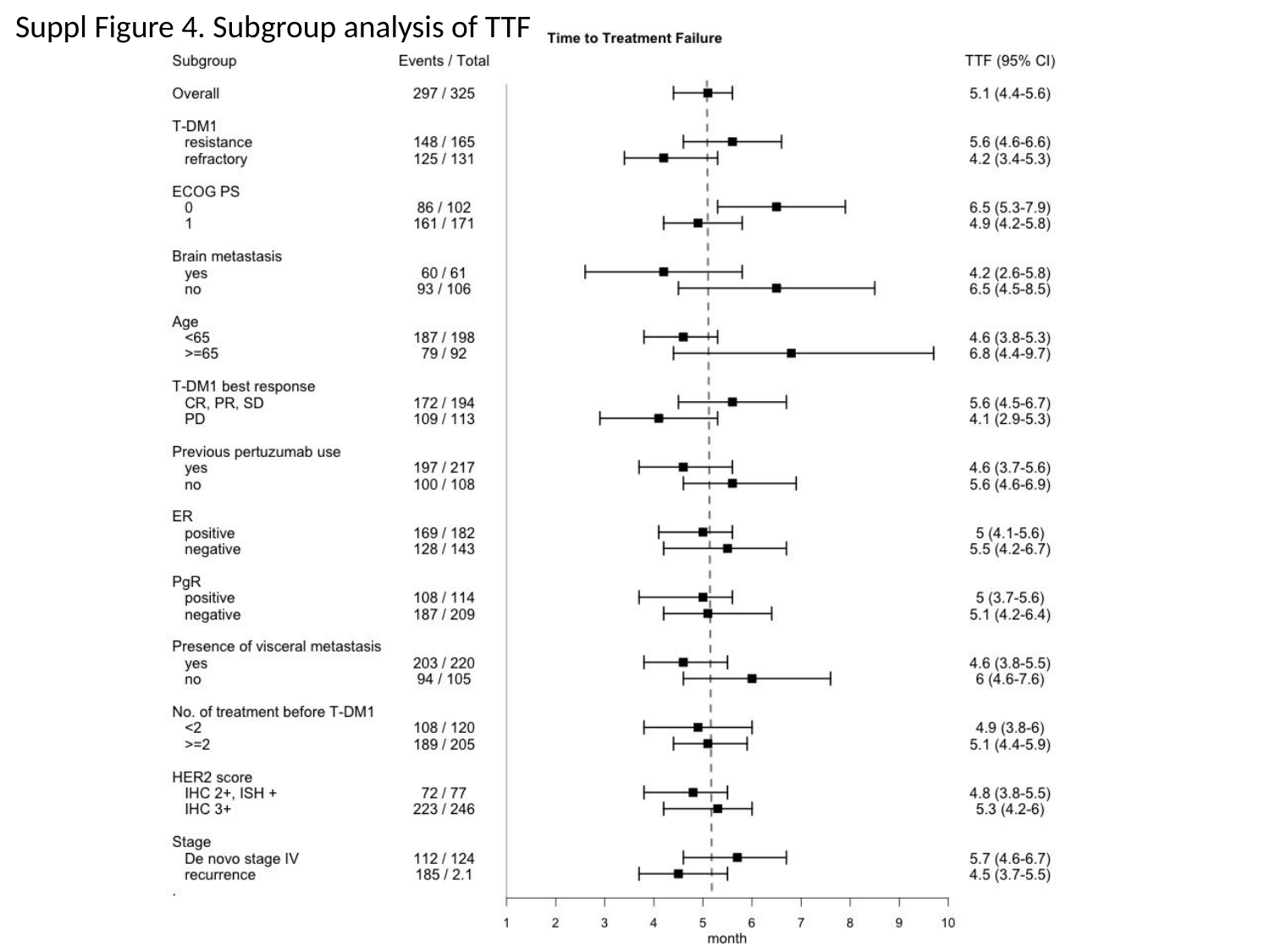

Suppl Figure 4. Subgroup analysis of TTF

## Slide 7
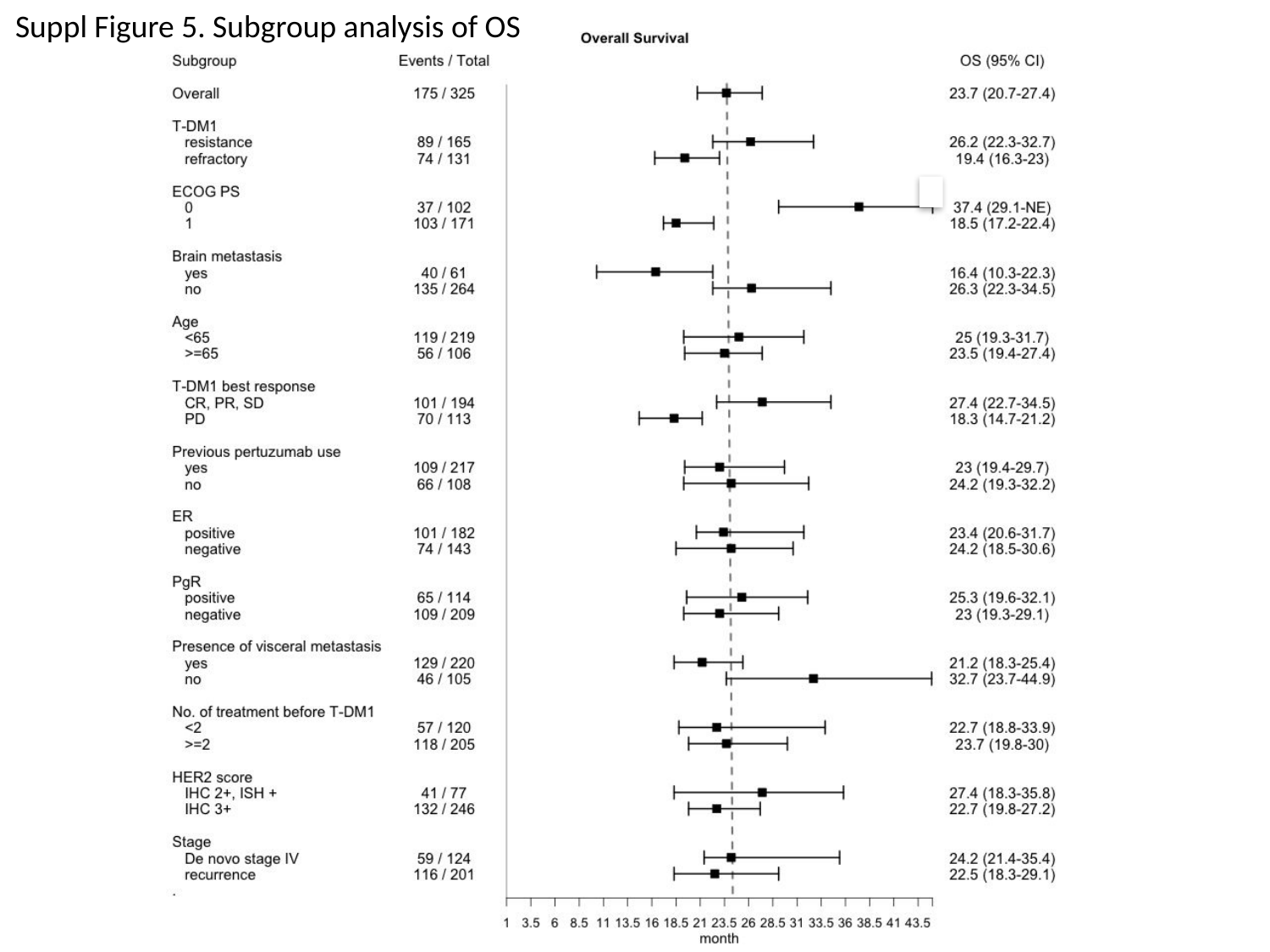

Suppl Figure 5. Subgroup analysis of OS
