# Supplementary material for: Clinical benefit of treatment after trastuzumab emtansine for HER2-positive metastatic breast cancer: a real-world multi-centre cohort study in Japan (WJOG12519B)
Source: Breast Cancer. 2021 Jan 2;28(3):581–91. doi: 10.1007/s12282-020-01192-y (PMC8064974; doi:10.1007/s12282-020-01192-y)
Supplement: Supplementary file 2 — Supplementary file2 (DOCX 19 KB) [file 12282_2020_1192_MOESM2_ESM.docx]

**Supple Table 1. ORR and DCR of T-DM1 (n = 290)**

| Characteristics | Number of cases (n= 290) | % |
| --- | --- | --- |
| Duration of T-DM1 treatment |  |  |
| Median number of doses (min - max) | 7 | (1-61) |
| Best response of T-DM1 * |  |  |
| CR | 3 | 1.0% |
| PR | 68 | 23.4% |
| SD | 104 | 35.9% |
| PD | 101 | 34.8% |
| NE | 14 | 4.8% |
| ORR | 71 | 24.5% |
| DCR | 175 | 60.3% |
| Reason for terminating T-DM1 |  |  |
| Progressive disease | 288 | 88.6% |
| Other reasons | 37 | 11.4% |

* Patients with target lesion (n = 290). Abbreviations: CR, complete response; PR, partial response; SD, stable disease; PD, progressive disease; NE, not evaluable; ORR, overall response rate; DCR, disease control rate

**Supple Table 2. Types of Post T-DM1 treatment in detail in descending order (n = 325)**

|  | Number of cases (n = 325) | % |
| --- | --- | --- |
| lapatinib + capecitabine | 63 | 19.4 |
| trastuzumab + pertuzumab + taxane | 47 | 14.5 |
| trastuzumab + pertuzumab + eribulin | 25 | 7.7 |
| trastuzumab + eribulin | 21 | 6.5 |
| trastuzumab + vinorelbine | 21 | 6.5 |
| trastuzumab + pertuzumab + vinorelbine | 19 | 5.8 |
| anthracycline | 17 | 5.2 |
| trastuzumab + gemcitabine | 13 | 4 |
| trastuzumab + capecitabine | 12 | 3.7 |
| eribulin | 11 | 3.4 |
| trastuzumab | 11 | 3.4 |
| trastuzumab + HTx | 10 | 3.1 |
| trastuzumab + taxane | 9 | 2.8 |
| taxane | 7 | 2.1 |
| lapatinib + HTx | 6 | 1.8 |
| trastuzumab + pertuzumab + gemcitabine | 6 | 1.8 |
| HTx | 6 | 1.8 |
| others | 21 | 6.5 |

HTx, hormone therapy.
